# Bidirectional modulation between infiltrating CD3^+^ T-lymphocytes and astrocytes in the spinal cord drives the development of allodynia in monoarthritic rats

**DOI:** 10.1038/s41598-017-18357-z

**Published:** 2018-01-08

**Authors:** Ya-Lan Zhou, Shu-Zhuan Zhou, Hao-Ling Li, Man-Li Hu, Hui Li, Qing-Huan Guo, Xiao-Ming Deng, Yu-Qiu Zhang, Hua Xu

**Affiliations:** 1 Department of Anesthesiology, Changhai Hospital, The Second Military Medical University, Shanghai, 200433 China; 2 Institutes of Brain Science and State Key Laboratory of Medical Neurobiology, Collaborative Innovation Center for Brain Science, Fudan University, Shanghai, 200032 China

## Abstract

Increasing evidence suggests that T cells and glia participate in the process of neuropathic pain. However, little is known about the involvement of T cells or the interaction between glia and T cells at the molecular level. Here we investigated the phenotype of T cell infiltration into the spinal cord in inflammatory pain and explored potential crosstalk between glia and T cells. The establishment of monoarthritis produced T cell infiltration and astrocyte activation, exhibiting similar kinetics in the spinal cord. T-cell-deficient (Rag1^−/−^) mice significantly attenuated MA-induced mechanical allodynia and GFAP upregulation. Double immunofluorescence staining showed that CD3 mainly colocalized with interferon-gamma (IFN-γ). Western blot and flow cytometry showed that multiple intrathecal administrations of astrocytic inhibitor fluorocitrate decreased IFN-γ-production without decreasing T cell number in the spinal cord. Spinal IFN-γ blockade reduced MA-induced mechanical allodynia and astroglial activation. In contrast, treatment with rIFN-γ directly elicited persistent mechanical allodynia and upregulation of GFAP and pJNK1/2 in naïve rats. Furthermore, rIFN-γ upregulated the phosphorylation of NF-κB p65 in cultured astrocytes *vitro* and spinal dorsal horn *vivo*. The results suggest that Th1 cells and astrocytes maintain inflammatory pain and imply that there may be a positive feedback loop between these cells via IFN-γ.

## Introduction

In clinical pathological pain, arthritic pain is a major health care burden. Arthritis affects 1% of the worldwide population and initiates with inflammation of the synovium in peripheral joints, which progresses to destruction of articular cartilage, leading to significant joint degeneration, pain and loss of function^1,2^. However, because of the incomplete understanding of pathophysiological mechanisms underlying the development of chronic pain, pharmacologic and non-pharmacologic therapies available at present are unable to provide definite therapeutic effects.

It is now well established that neuroinflammation is the critical mechanism in central sensitization (i.e. enhanced responses of pain circuits in the spinal cord and brain). Glial cells, which are mainly involving microglia and astrocytes, interact with neurons to comprise the neuroimmune sensory mechanism. Microglia are immune-component cells of the central nervous system (CNS), rapidly activated in response to inflammatory insult, lead to the increased production neuromodulators (eg. TNF-α, IL-1β, IL-18, and brain-derived growth factor), which enhances pain signal transmission, mainly contributing to the generation of pain^3,4^. While astrocytes are the most abundant glial cells, mainly participating in the progression of chronic pain by upregulation of Cx43, CCL2, MMP-2, etc.^3,4^.

Increasing evidence suggests that spinal cord-infiltrating T lymphocytes contributes to the development and maintenance of neuropathic pain^5–7^. It has been reported that in both animals and humans, peripheral nerve injury does not induce pain in newborns compared with that in adults^5,8^. Comparison of the microarray gene profiles of the spinal cord from neonate and adult animals 7 days post-spared nerve injury identified 148 differentially expressed genes. Most of these genes were related to immune function, and they included numerous genes involved in T-cell signaling^5^. Several studies have shown that in neuropathic pain models (e.g. spinal nerve L4 or L5 transection, CCI) T cells infiltrated into the dorsal horn ipsilateral to the surgery site. In the following phenotype identification, infiltrating CD4^+^ T lymphocytes expressed a pro-inflammatory Th1^7,9,10^. While reducing on T cell number by gene knockout or splenectomy induced less neuropathic mechanical allodynia and that infusion of Th1 cells can restore pain hypersensitivity in T cell-deficient animal^9,10^. However, the role of T cells in inflammatory pain and the involved regulatory mechanisms are unclear.

In the present study, we investigated the subtypes and functional roles of T cells infiltrating the spinal cord of rats with complete Freund’s adjuvant (CFA)-induced monoarthritis (MA), a model of inflammatory pain. We also examined the molecular crosstalk between glia and T cells in the CNS after inflammatory insult.

## Results

### Monoarthritis induces upregulation of infiltrating T cell number and activation of glia in the spinal cord

**Figure 1:**
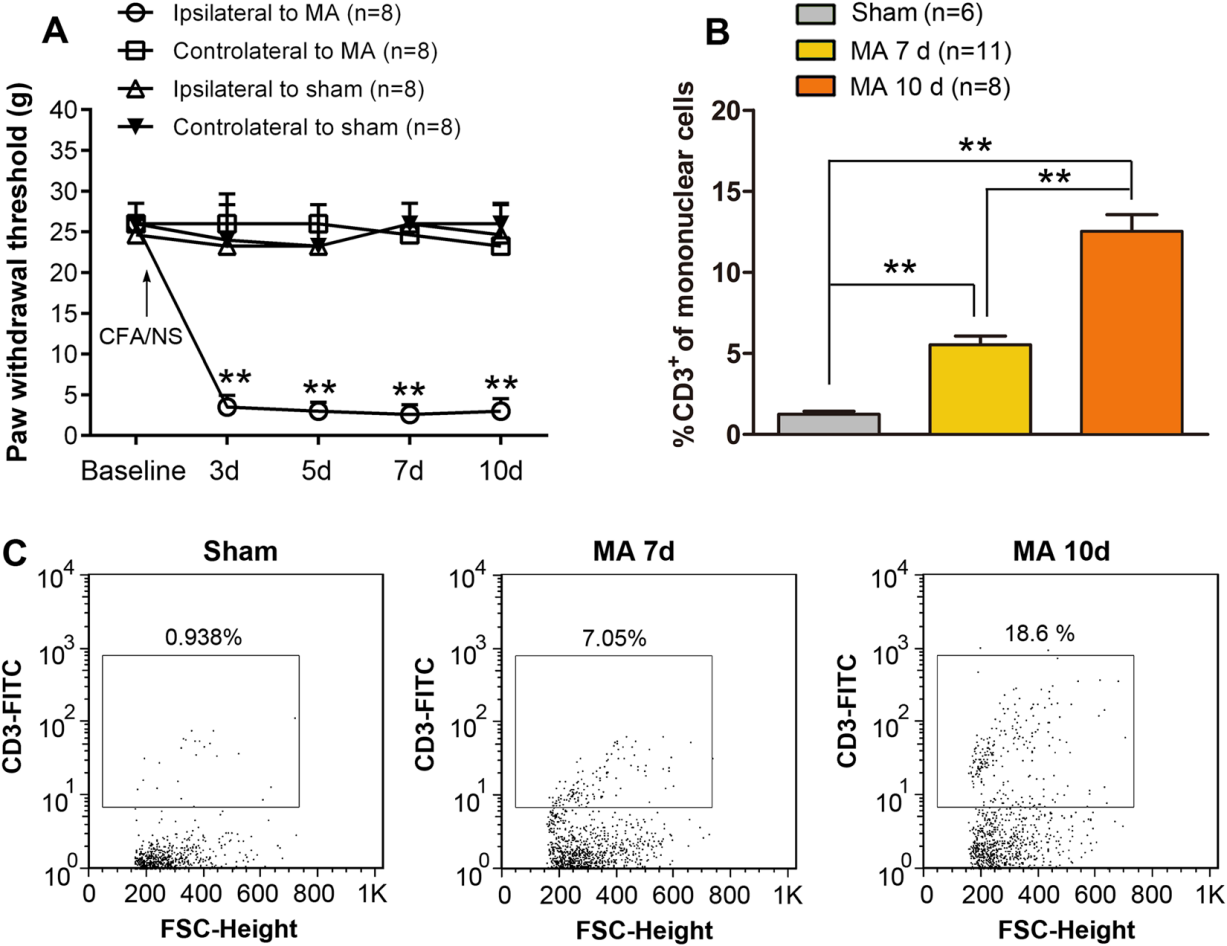
Mechanical allodynia and T cell infiltration into the spinal cord after monoarthritis (MA). (**A)** Time course of paw withdrawal threshold (PWT) of the ipsilateral (Ipsi) and contralateral (Cont) hind paws of rats with MA and sham rats; **p < 0.01 indicates significant difference between rats with MA (Ipsi) and sham rats (Ipsi) (n = 8 per group). **(B,C)** Results of flow cytometric analysis showing the percentage of CD3-positive cells among mononuclear cells obtained from the lumbar spinal cord; **p < 0.01.

Sprague–Dawley rats showed obvious joint inflammation (edema and erythema) within 24 h after CFA injection. The paw withdrawal threshold (PWT) of the ipsilateral hind paw to the MA-affected limb declined to a minimum by day 3 and remained at this level throughout the study (Two-way ANOVA; treatments: F_3,28_ = 403.0, p < 0.001; treatment × time: F_12,112_ = 20.80, p < 0.001). In contrast, no significant change of mechanical threshold and ankle inflammation was observed in the contralateral hind paw to the MA-affected limb or the bilateral hind paws of sham rats (Fig. 1A). MA-induced mechanical allodynia persisted for at least 10 days in the affected limb, and thus, rats with MA are well adequate for pathological pain research.

To detect spinal cord-infiltrating T cells, we examined CD3^+^ T lymphocytes in the lumbar spinal cord on days 7 and 10 after MA via flow cytometry with antibody that identify the CD3 (a common marker for T cells). Flow cytometric analysis showed a significant increase in the number of spinal cord-infiltrating T cells on day 7, and the increase became more pronounced on day 10 after the inflammatory insult (One-way ANOVA, F_2,22_ = 55.27, p < 0.001). No significant migration of T cells into the spinal cord was found in sham rats (Fig. 1B and C).

**Figure 2:**
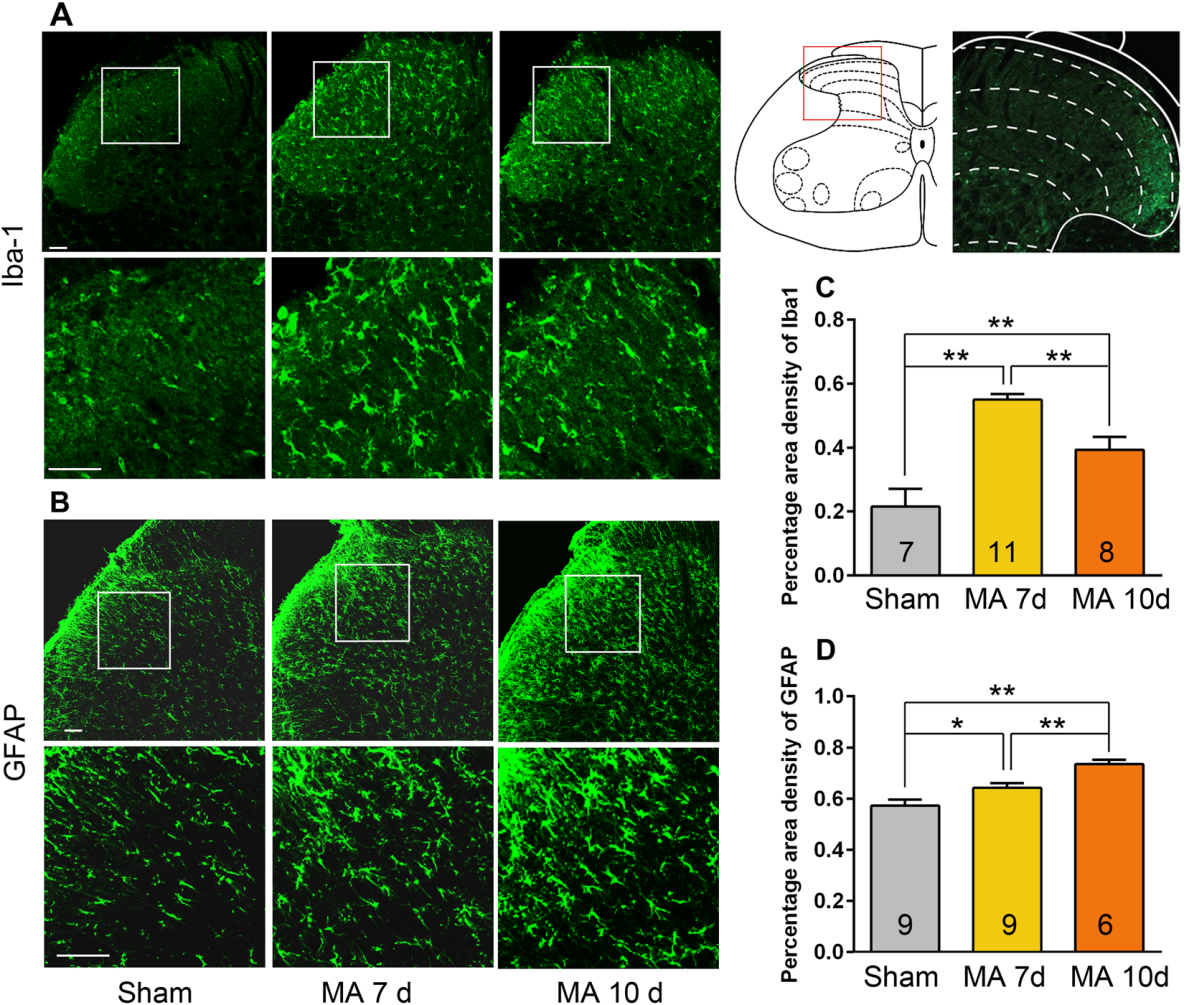
MA induced microglia and astrocytes activation in the spinal cord. (**A,C)** Increased percentage area density of Iba1 (a microglial marker) in the ipsilateral lumbar spinal dorsal horn of rats with MA; **p < 0.01. **(B,D)** Immunohistochemical analysis showing a marked increase in GFAP (an astrocytic marker) immunoreactivity in the ipsilateral spinal dorsal horn on days 7 and 10 in rats with MA; *p < 0.05, **p < 0.01. Scale bars = 50 μm.

A robust activation of microglia was observed on days 7 and 10 post-MA. Activated microglia were present as large cell bodies with short or thick processes. On days 7 and 10, the percentage of ionized calcium binding adapter molecule-1 (Iba1)-positive area in the spinal dorsal horn of MA rats was higher than that in sham ones (One-way ANOVA, F_2,23_ = 21.53, p < 0.001). However, microglial activation gradually weakened. In rats with MA, Iba1 expression was significantly lower on day 10 than that on day 7 post-MA (One-way ANOVA followed by post-hoc Student–Newman–Keuls test, q = 4.493, p < 0.01; Fig. 2A and C).

The time-course of astrocytic activation was similar to infiltrating T cells in the spinal dorsal horn. Activated astrocytes exhibited intense glia fibrillary acidic protein (GFAP) immunoreactivity and appeared hypertrophied with thick processes (Fig. 2B and D). Significant elevation of the percentage area density of GFAP was observed at days 7 and 10 post-MA (One-way ANOVA, F_2,21_ = 12.99, p = 0.0002) with the more prominent increase being on day 10 (One-way ANOVA followed by post-hoc Student–Newman–Keuls test, q = 4.137, p < 0.01; Fig. 2B and D). The data indicate that astrocytic activation progressively increased from day 7 to at least day 10.

### T cell activation in the spinal dorsal horn of rats with MA

Pain arising from MA increased the number of T cells in the spinal cord, which was most likely associated with the observed immunological reaction. Therefore, we examined T cell activation and subpopulations by performing double immunostaining of T cell-specific cytokines with cell-specific markers, including CD3 for T cells, Iba1 for microglia and GFAP for astrocytes on sections of lumbar spinal cord from rats at day 10 after CFA injection.

**Figure 3:**
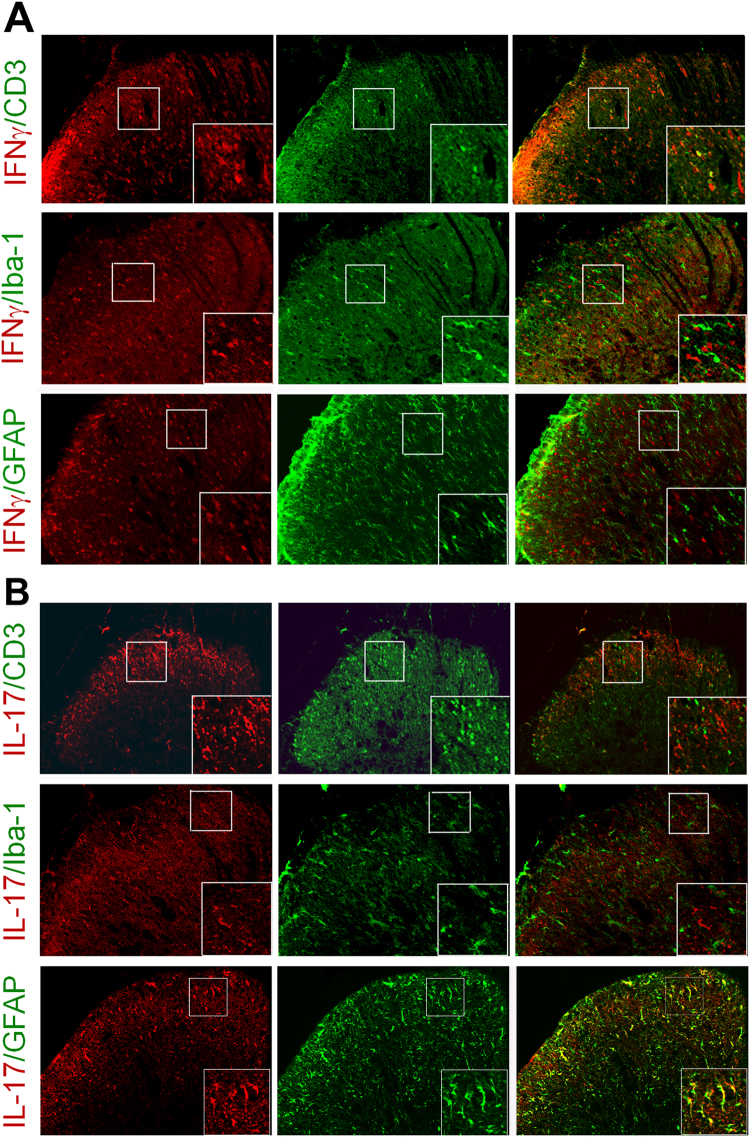
Expression of IFN-γ and IL-17 in the spinal cord. (**A)** Results of immunofluorescence staining showing that IFN-γ colocalizes with CD3 and that **(B)** IL-17 colocalizes with GFAP. Scale bars = 50 μm.

On day 10 after MA, immunofluorescence staining showed that CD3-positive T cells mainly distributed in the ipsilateral superficial spinal dorsal horn (laminae I–III; Fig. 3A). Interferon-gamma (IFN-γ), the signature cytokine of Th1 cells, was expressed in the ipsilateral superficial spinal dorsal horn and colocalized with CD3, but did not with Iba1 and GFAP (Fig. 3A). However, very few interleukin-17 (IL-17), the signature cytokine of Th17 cells, coexpressed with CD3. Instead, IL-17-immunoreactive cells were mainly restricted to GFAP-positive areas but not in Iba1-positive sites (Fig. 3B). These data indicate Th1 cell is a predominant infiltrating T cell subtype in the spinal cord after inflammatory insult.

### Effects of glial inhibitors on infiltrating T cells in the spinal cord

**Figure 4:**
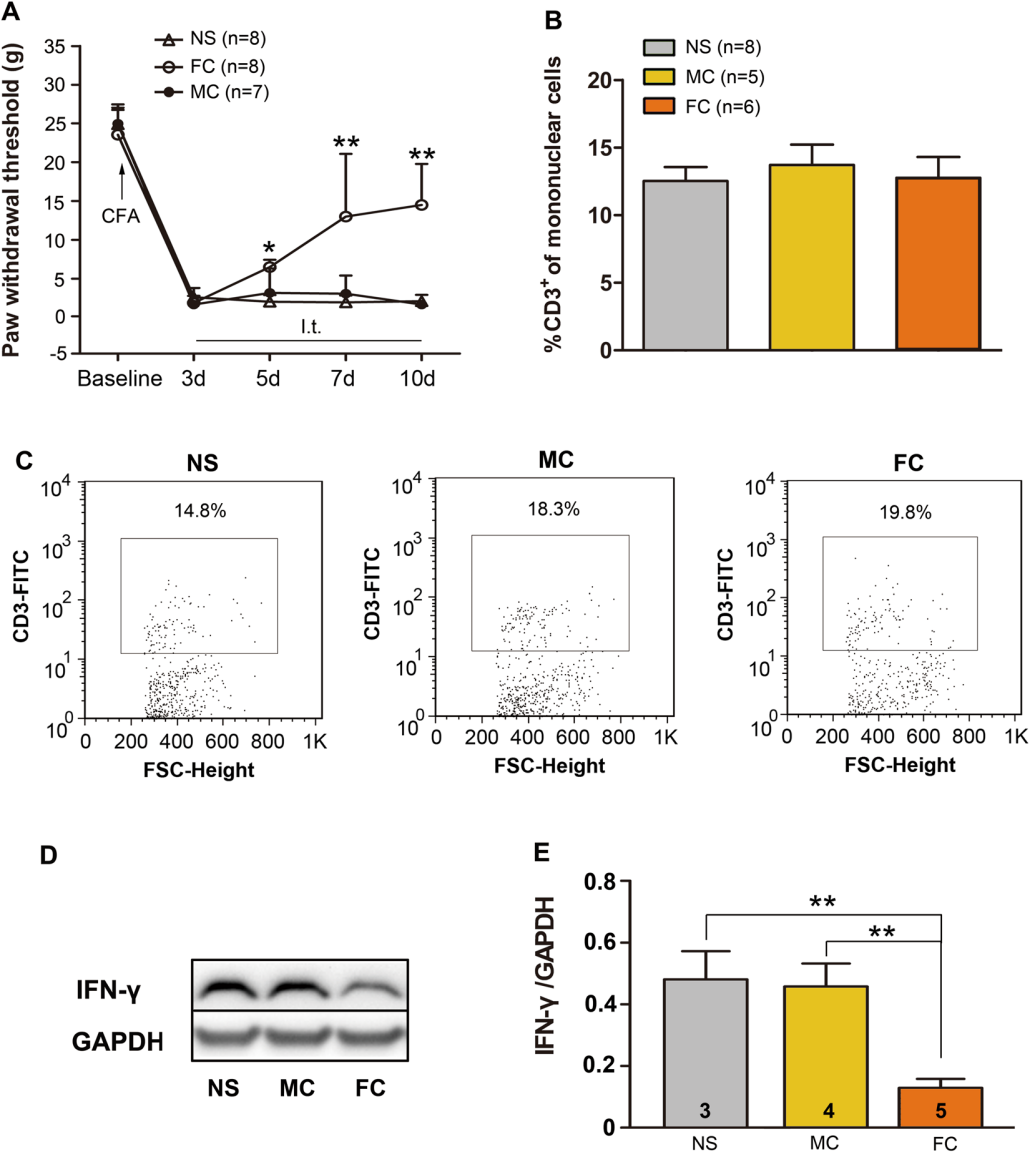
Effect of intrathecal injection of glial inhibitors on T cell number and capacity. (**A)** Mechanical allodynia was improved in fluorocitrate (FC, an astrocytic inhibitor)-treated rats compared with that in NS-treated rats; *p < 0.05 and **p < 0.01, FC vs NS group (n = 7–8). **(B,C)** Unaltered number of T cells infiltrating into the spinal cord. **(D,E)** Decreased IFN-γ expression after the intrathecal administration of FC; **p < 0.01.

To determine the possible impact of the activation of microglia and astrocytes on T cells in the spinal cord, minocycline (100 μg), a microglial inhibitor, and fluorocitrate (1nmol), a nonselective glial inhibitor were intrathecally (i.t.) administered once daily for consecutive 8 days by 3 d after MA. Consistent with previous studies^11,12^, minocycline-treated MA rats showed no significant improvement in the PWT. In contrast, fluorocitrate-treated MA rats showed significantly alleviated mechanical hypersensitivity (Two-way ANOVA, treatments: F_2,20_ = 21.55, p < 0.001; treatment × time: F_8,80_ = 13.41, p < 0.001; Fig. 4A). However, flow cytometric analysis with anti-CD3 antibody showed no significant differences in the relative percentage of T cells in the spinal cord among minocycline-, fluorocitrate-, and NS-treated rats (Fig. 4B and C).

Besides the T cell number, the ability of cytokine production directly affects the immunological effect. Next, we investigated the functional capacity of T cells after treatment with glial inhibitors by measuring the production of proinflammatory cytokines, with an emphasis on IFN-γ production. We performed western blotting to semiquantitatively measure IFN-γ expression. Multiple administration of fluorocitrate significantly reduced the IFN-γ level in the spinal cord compared with that in NS-treated rats (One-way ANOVA, F_2,9_ = 7.926, p = 0.0104; Fig. 4D and E). However, the ability of infiltrating T cells to produce IFN-γ was not significantly changed in minocycline-treated group compared with that in NS-treated rats, indicating that the function of Th1 cells is modulated by astrocytes, but not microglia, in inflammatory pain.

### Reduction of mechanical allodynia and GFAP level in Rag1^−/−^ mice with MA

**Figure 5:**
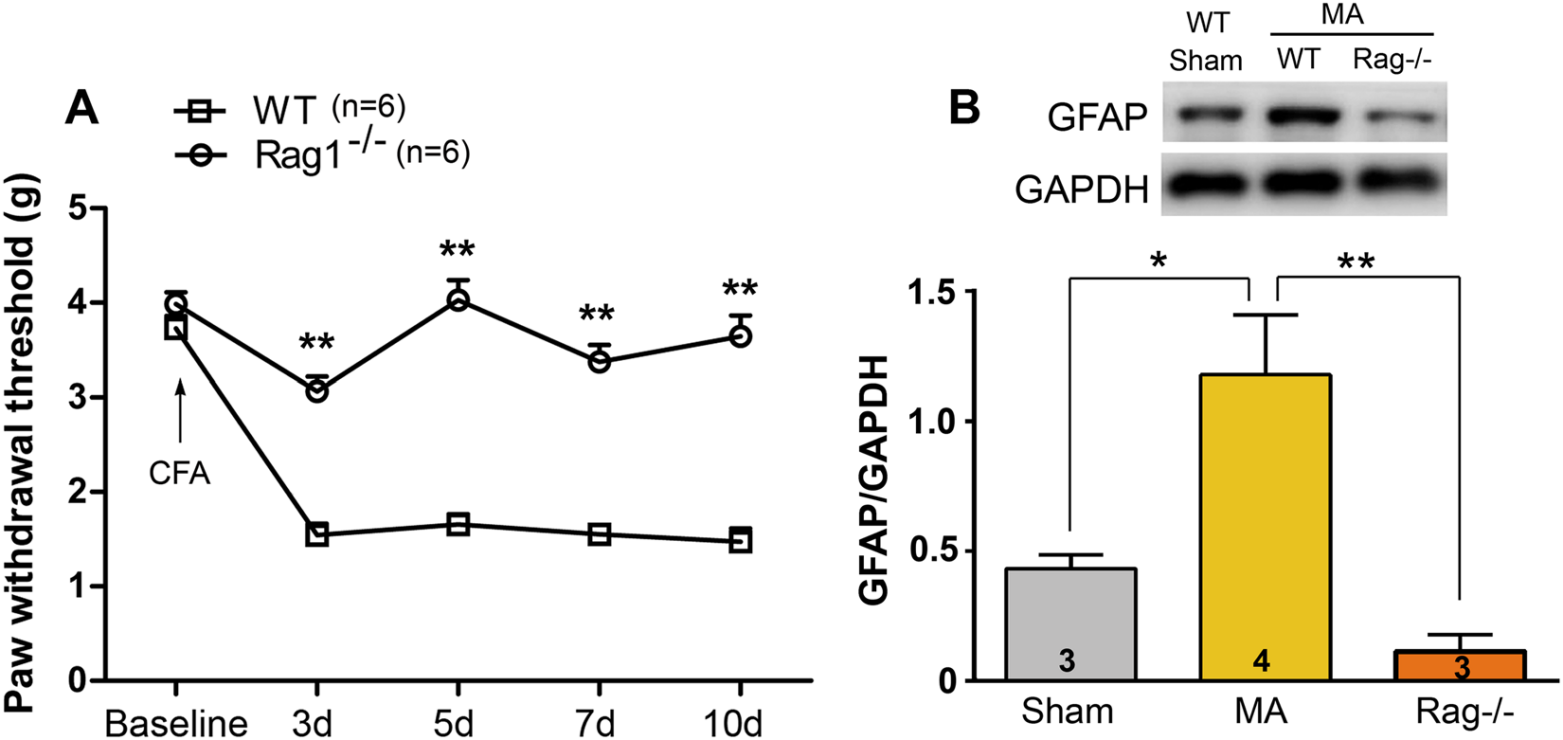
Mechanical pain threshold and GFAP expression in Rag1^−/−^ mice following CFA injection. (**A)** T-lymphocyte-deficient attenuated mechanical allodynia; **p < 0.01, Rag1^−/−^ mice vs wild-type littermate control (n = 6). **(B)** T-lymphocyte-deficient eliminated MA-induced GFAP upregulation; *p < 0.05, **p < 0.01.

T cell infiltration into the spinal cord of rats with MA may be implicated in mechanical allodynia. And therefore, we measured mechanical pain threshold in a T-lymphocyte-deficient mouse strain, the Rag1^−/−^ on a C57BL/6j background. There was no significant difference between the Rag1^−/−^ and wild-type littermate controls in mechanical pain hypersensitivity before MA establishment. However, Rag1^−/−^ mice displayed less mechanical allodynia from days 3 to 10 post CFA injection, compared with the controls (Two-way ANOVA, treatments: F_1,10_ = 325.3, p < 0.001; treatment × time: F_4,40_ = 14.15, p < 0.001; Fig. 5A).

It is well known that astrocytes activation is correlated with hyperalgesia. We, therefore, examined spinal GFAP expression following CFA-MA in Rag1^−/−^ mice. On day 10 post-MA, GFAP expression was significantly upregulated in wild-type mice. However, a significant difference was observed between the Rag1^−/−^ and wild-type littermate controls in spinal GFAP expression (One-way ANOVA, F_2,7_ = 10.84, p = 0.0072; Fig. 5B).

### Blockade of IFN-γ attenuates MA-induced pain and reduces astroglial activation

**Figure 6:**
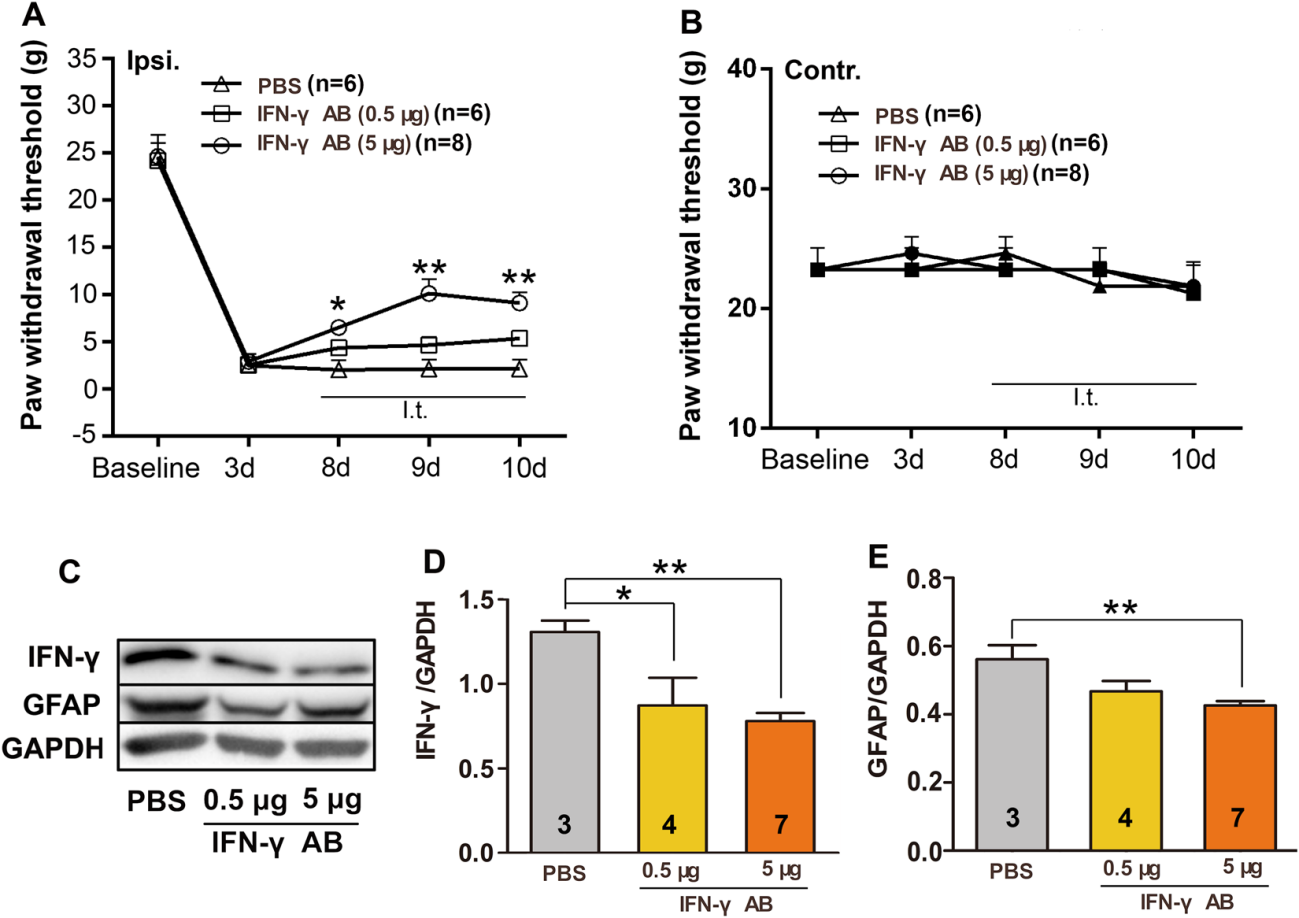
Effect of intrathecal injection of anti-IFN-γ antibody (IFN-γ AB) on MA-induced allodynia. (**A)** Treatment with IFN-γ AB (5 μg) attenuated mechanical allodynia; *p < 0.05 and **p < 0.01, IFN-γ AB (5 μg) vs PBS-injected control (n = 6–8). **(B)** No obvious change was observed in the PWT of the contralateral hind paw of rats with MA after the intrathecal injection of 0.5 and 5 μg IFN-γ AB. **(C,D)** Results of western blotting showing decreased IFN-γ immunoreactivity in the ipsilateral spinal dorsal horn after IFN-γ blockade; *p < 0.05, **p < 0.01. The cropped bands had been run under the same experimental conditions. **(C,E)** Decreased GFAP expression in the ipsilateral spinal dorsal horn after IFN-γ blockade; **p < 0.01.

In the spinal cord of rats with MA, IFN-γ expressed by Th1 cells may play an important role in the maintenance of mechanical hypersensitivity. Therefore, we hypothesized that IFN-γ inhibition in the spinal cord attenuated MA-induced pain and reduced astrocytes activation. Anti-IFN-γ antibodies (5 μg) were i.t. injected on days 7, 8, 9 and 10 after MA. We observed that inhibition of IFN-γ significantly reversed the established mechanical allodynia on the ipsilateral hind paw after the stimulation of inflammation (Two-way ANOVA, treatments: F_2,17_ = 9.758, p < 0.001; treatment × time: F_8,68_ = 4.981, p < 0.001; Fig. 6A). However, intrathecal administration of anti-IFN-γ antibody (0.5 μg) produced minor, but no significant change, in the threshold on the MA-affected hind paw. In contrast, pain threshold on the contralateral hind paw did not vary significantly from baseline values throughout the study (Fig. 6B).

We also observed that multiple treatments with IFN-γ-neutralizing antibody (0.5 or 5 μg) significantly reduced the MA-induced upregulation of IFN-γ in the spinal cord (One-way ANOVA, F_2,11_ = 7.396, p = 0.0092; Fig. 6C and D). In addition, multiple intrathecal injections of 5 μg of anti-IFN-γ antibody decreased GFAP level in the ipsilateral spinal dorsal horn of rats with MA (One-way ANOVA, F_2,11_ = 5.327, p = 0.0241; Fig. 6C and E). However, treatment with 0.5 μg of anti-IFN-γ antibody made no significant change in the expression of GFAP.

### Exogenous IFN-γ induces mechanical hyperalgesia and astrocytic activation

**Figure 7:**
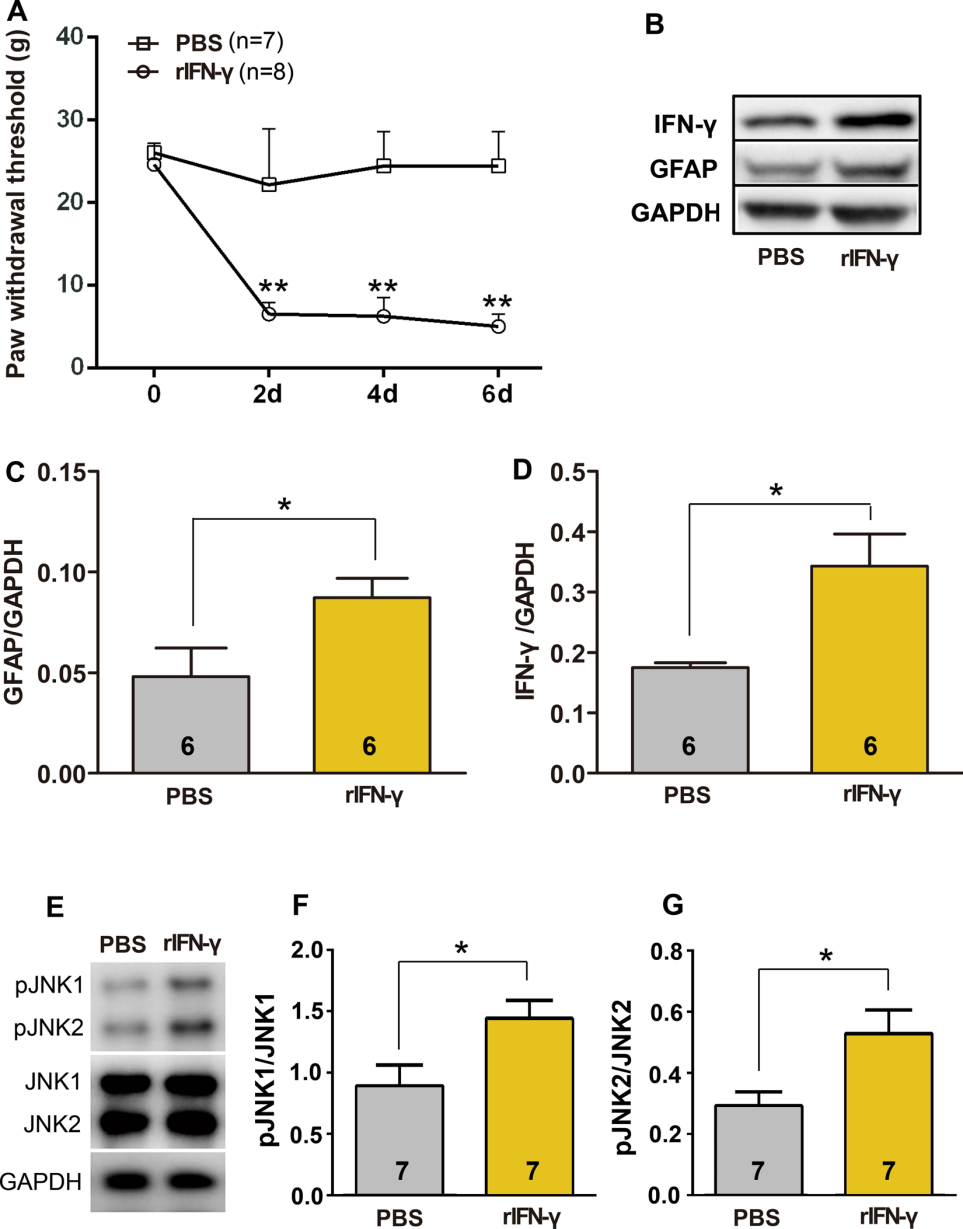
Allodynia induction and astrocyte activation by recombinant IFN-γ (rIFN-γ) injection. (**A)** Compared with intrathecal injection of PBS, intrathecal injection of rIFN-γ induced mechanical allodynia; **p < 0.001, rIFN-γ vs PBS (n = 7–8). **(B–D)** Upregulation of GFAP (**B**,**C**) expression and IFN-γ (**B**,**D**) after intrathecal injection of rIFN-γ; *p < 0.05. **(E–G)** Upregulation of pJNK1 (**E**,**F**) and pJNK2 (**E**,**G**) after intrathecal injection of rIFN-γ; *p < 0.05.

To further test the effect of IFN-γ on behavioural response to mechanical stimulation, we selected a similar treatment regimen, as described previously, to mimic the prolonged presence of IFN-γ in the CNS during a diseased state^13^. Briefly, recombinant IFN-γ (rIFN-γ) was i.t. administrated every 2 days (in all, 4 injections per animal). Behaviour assessment was performed before every i.t. injection. We observed that a single delivery of exogenous IFN-γ into the spinal cord directly elicited mechanical hypersensitivity for at least 48 h, and multiple administration of rIFN-γ produced persistent allodynia (Two-way ANOVA, treatments: F_1,13_ = 166.2, p < 0.001; treatment × time: F_3,39_ = 27.11, p < 0.001; Fig. 7A). The spinal cord on day 7, was collected for western blotting. Western blotting results showed increased IFN-γ immunoreactivity (Student’s *t*-test, t_0.05/2,10_ = 3.110, p = 0.0111; Fig. 7B and D). In addition, GFAP expression was significantly upregulated in exogenous IFN-γ-treated rats compared with that in phosphate-buffered saline (PBS)-treated rats (Student’s *t*-test, t_0.05/2,10_ = 2.270, p = 0.0466; Fig. 7B and C).

JNK, especially JNK1 in spinal astrocytes is functional, taking part in the persistence of inflammatory pain after CFA Intraplantar injection^14,15^. rIFN-γ was i.t. administrated at dose of 1000 U once. The spinal cord was collected 10 min after rIFN-γ for western blotting analysis. The levels of total JNK1 and JNK2 did not change after rIFN-γ treatment (Fig. 7E–G). In contrast, rIFN-γ incuced a rapid increase in pJNK1/JNK1 (Student’s t-test, t_0.05/2,9_ = 2.476, p = 0.0352; Fig. 7E and F) and pJNK2/JNK2 levels (Student’s t-test, t_0.05/2,7_ = 2.661, p = 0.0324; Fig. 7E and G).

### IFN-γ increases the phosphorylation of nuclear factor-κB p65 (p-NF-κB p65) *in vitro* and *vivo*

**Figure 8:**
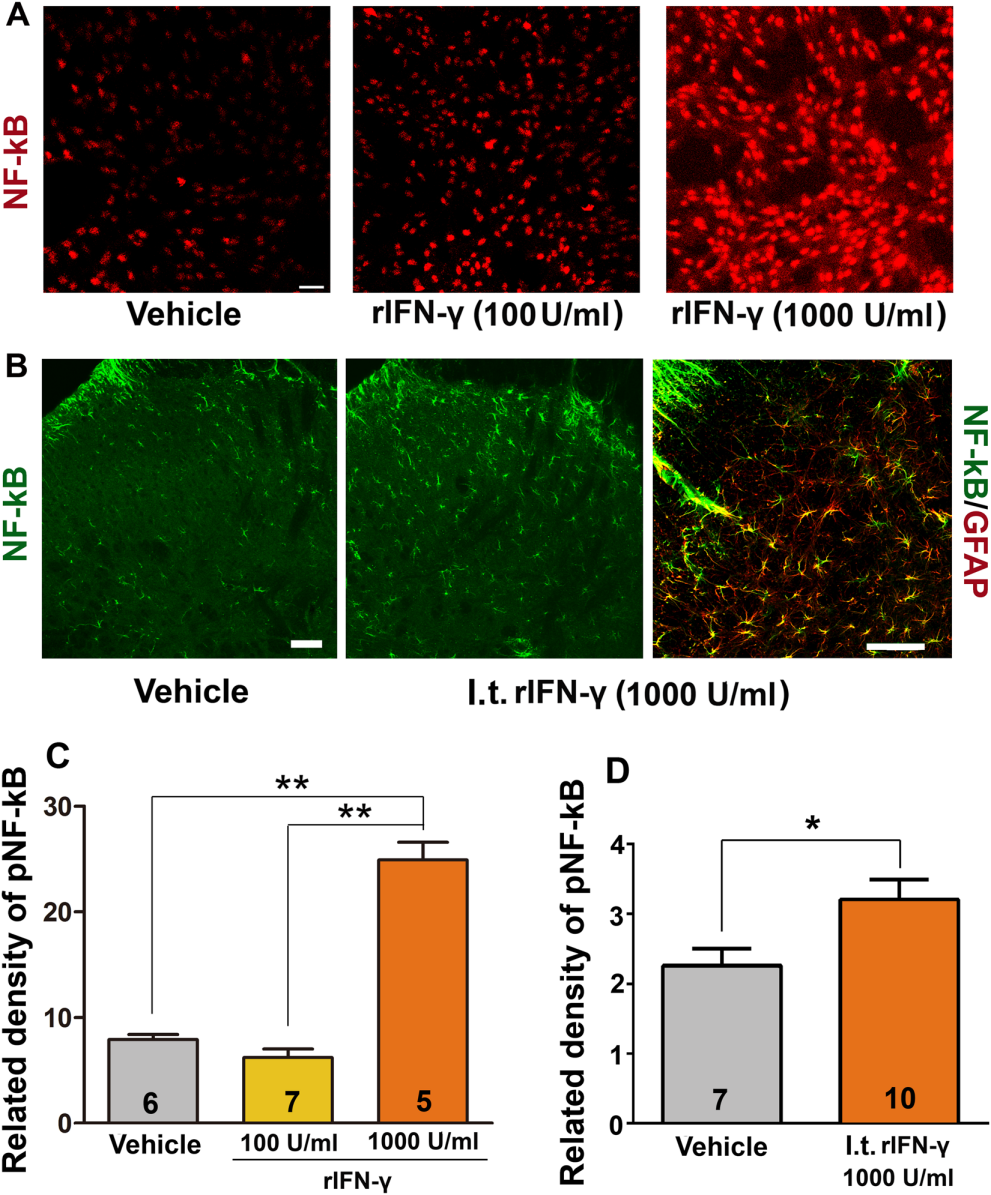
Effect of IFN-γ on the phosphorylation of NF-κB p65 (p-NF-κB p65). (**A,C)** p-NF-κB p65 was upregulated in 1000 U/ml rIFN-γ-treated culture astrocytes compared with that in vehicle control and 100 U/ml rIFN-γ-treated cells; **(B,D)** I.t. injection of 1000 U/ml rIFN-γ significantly increased p-NF-κB p65 expression level in the spinal dorsal horn. **p < 0.01, *p < 0.05; Scale bars = 50 μm.

Activation of the NF-κB pathway is correlated with behavioural hyperalgesia and is of interest for studying astrocytic function because numerous genes encoding inflammation- and pain-related molecules are controlled by NF-κB^16^. Post-translational modification of p65, an NF-κB subunit, significantly contributes to the transactivation potential of NF-κB^17^. Phosphorylation of p65 at Ser536 is important for its transactivation function. Therefore, we examined the role of IFN-γ in the phosphorylation of NF-κB p65 in primary cultured astrocytes by performing immunofluorescence staining of p-NF-κB p65 with anti-p-NF-κB p65 antibody specific to Ser536. We observed that NF-κB p65 phosphorylation was significantly upregulated in cells treated with 1000 U/ml IFN-γ for 48 h compared with that in control cells (One-way ANOVA, F_2,15_ = 95.50, p < 0.001; Fig. 8A and C). However, treatment with 100 U/ml IFN-γ did not affect the phosphorylation of NF-κB p65 in astrocytes.

To determine whether the NF-κB was also actived by IFN-γ in the spinal cord *in vivo*, recombinant IFN-γ (rIFN-γ) was i.t. administrated every 2 days (in all, 4 injections per animal) as behavioral test. After the last treatment, immunofluorescence staining was applied. The increased NF-κB p65 phosphorylation was observed in rIFN-γ treatment rats (Student’s *t*-test, t_0.05/2,31_ = 2.138, p = 0.0405; Fig. 8B and D). pNF-κB p65 immunoreactivity was almost expressed in GFAP positive cells (Fig. 8B).

## Discussion

In this study, we examined whether T cell-mediated adaptive immune response was involved in the development of inflammatory pain. First, we observed that CD3^+^ T-lymphocytes migrated into the spinal cord, and involved in chronic inflammatory pain. Second, our results showed that spinal IFN-γ-producing Th1 cells maintained inflammatory pain at least in part by accelerating astroglial activation. Third, we confirmed that astrocytes played an important role in the development of MA-induced pain and that this role was partially mediated by the modulation of Th1 cell-associated IFN-γ expression in the spinal dorsal horn.

To date, limited studies have investigated the immune inflammatory response induced by T cells during inflammatory pain. Moreover, most of these studies have focused on neurological diseases such as multiple sclerosis (MS). T cells differentiate into Th1 and Th17 cells, the two proinflammatory phenotypes critical for the pathogenesis of MS. Approximately 80% patients with MS experience chronic pain^18–20^. Here we presented evidence that T cells contribute to inflammatory pain induced by MA. We showed that T-cell-defficient male mice developed less mechanical pain hypersensitivity than wide type controls after MA, consistent with the observation of Moalem *et al*. in CCI-induced neuropathic pain^10^. Furthermore, we demonstrated that MA-induced upregulation of spinal GFAP in wild-type mice failed to occur in Rag1^−/−^ mice, suggesting spinal astrocytes may also be a site of action of T cells. Interestingly, a recent report indicated male mice preferentially depended on microglia to produce allodynia after injury, and the female inclined to use adaptive immune cells instead of microglia^21^. This study suggested that besides the differences in pain models and species or strains, gender differences are important factors affecting pain.

Consistent with previous studies on MS, we observed that CD3^+^ T cells were present in the spinal cord of rats with MA. One study on MS showed that the above two T cell subtype depended on different integrins in a differential manner in migrating into the CNS and Th17 cells entered supraspinal parts of CNS^19^. Our results showed that the T cell marker mainly colocalized with IFN-γ and barely colocalized with IL-17, which are the hallmarks of Th1 and Th17 cells, respectively. This result suggests that Th1 cells may be the main subtypes involved in inducing chronic inflammatory pain. However, increasing evidence indicates that Th1 cells are involved in the development of pain after nerve injury, which is in contrast to that observed in the present study. After peripheral nerve injury, animals with IFN-γ-positive T cells in the spinal cord and those lacking T cells show attenuated mechanical allodynia, which can be reversed by transfusing Th1 cells from wild-type animals^6,9,22^.

In the present study, IL-17 was mainly present in the area of astrocytes on day 10 after CFA injection. This was consistent with the results of a study by Xianze Meng *et al*., which showed that IL-17 produced by astrocytes increased the levels of phosphorylated NR1 (p-NR1) to facilitate pain induction after subcutaneously injecting CFA into the plantar surface of rats^23^. P-NR1 is the key element that modulates the activity of *N*-methyl-d-aspartate receptor to induce pain.

Rats with MA exhibited reduced inflammatory hyperalgesia after intrathecal injection of anti-IFN-γ antibody. Further, intrathecal administration of IFN-γ elicited a lingering decreased PWT in naïve rats. These results suggest that Th1 cells contributed to inflammatory pain by producing IFN-γ. Previous electrophysiological studies have shown that IFN-γ induces neuronal hyperexcitability by directly acting on cognate receptors on dorsal horn neurons both *in vitro* and *in vivo* and that prolonged exposure to IFN-γ reduces GABAergic inhibition in the dorsal horn to induce pain^13,24,25^.

Astrocytes producing mediators (e.g., IL-1β and glutamate) generally play a critical role in maintaining pain^4^. However, it is unclear whether T cells infiltrating into the spinal cord affect astroglial activation. In this study, we observed GFAP downregulated in Rag1^−/−^ mice after MA injection. In consistence with this, Cao L, *et al*. observed decreased immunoreactivity to GFAP in mice lacking T cells after L5 spinal nerve transection^6^. In the following experiment, our results showed that IFN-γ supplementation also boosted GFAP expression and pJNK upregulation in naïve rats. In contrast, IFN-γ blockade suppressed astrocyte activation after inflammatory insult. The JNK pathway as an important functional pathway in persistent pain condition has been demonstrated. JNK activation after nerve injury is likely to regulate pain progression by accelerating spinal synthesis of inflammatory mediators such as nitric oxide, prostaglandin E2^14,15^. Further, phosphorylation of NF-κB p65 was upregulated after IFN-γ treatment both *in vivo* and *in vitro*. In our previous study, we have shown that the MA rats contained a markedly higher p-NF-κB p65 level in the spinal dorsal horn compared with that in the sham group on days 3, 7 post-operation^26^. The level of p-NF-κB p65 directly indicates the activity of the NF-κB pathway, which is correlated to pain hypersensitivity and is required for maintaining pain^4,17^. Thus, our data indicate that IFN-γ triggered pain at least in part by modulating astroglial activation. However, several studied showed that spinal microglia expressed IFN-γ receptor and pSTAT1, a molecule downstream of IFN-γ receptor activation^9,27^. IFN-γ induced microglia activation and hyperalgesia, which could be reduced by minocycline treatment, a microglia inhibitor^27^. Hence, whether activated microglia mediate the effects of IFN-γ on pain and GFAP expression still need to be studied.

Entry of Th1 cells into the CNS needs appropriate conditions that are most likely provided by activated microglia and astrocytes. Previous studies have shown that permeability of the blood-CNS barrier, which is a prerequisite for peripheral immune cells to infiltrate the CNS, increases in animals after inflammatory insult^28,29^. Alteration of the blood-CNS barrier permeability during pain maintenance cannot be reversed by blocking action potential^30^. Therefore, it is suggested that this alteration is induced by inflammatory mediators expressed by activated microglia and astrocytes^30^. In the present study, we observed extensive activation of microglia and astrocytes in the spinal cord of rats with MA. Compared to microglial activation, astrocyte proliferation progressed on days 7 and 10 after CFA injection, which was similar to that observed for T cells. Surprisingly, we observed no significant difference in the number of T cells in the spinal cord after inhibiting microglial and astroglial activation compared with that in control rats. However, it should be noted that this study focused on the chronic stage of pain. In particular, the interference of glial inhibitors began on day 3 post-MA after the emergence of pain hyperalgesia. At this time, the irreversible alteration of the blood-CNS barrier permeability may have already occurred. In addition, there is consensus that neurons exhibit immune-like nature, produce mediators (e.g., NO), and actively participate in pain generation. Studies in rats have shown that nociceptive signalling alters the molecular and functional properties of the blood-CNS barrier, thus augmenting its permeability^31^. Thus, inhibition of microglia or astrocytes was not sufficient to decrease the beneficial effect of the inflammatory microenvironment in the spinal cord on T cell infiltration. Therefore, the results of this study suggest that glial activation have no obvious contribution to T cell infiltration the spinal cord in the chronic phase of inflammatory pain. However, further research involving pretreatment with glial inhibitors or gene silencing should be performed to study the impact of glial activation on T cell infiltration during inflammatory pain.

We observed that the mean level of Th1 cell-related cytokine IFN-γ was decreased and that this alteration was accompanied with an improvement in mechanical allodynia after the suppression of astrocyte activation. Several studies have demonstrated that astrocytes function as non-professional antigen-presenting cells (APCs) and express cell surface MHC class II molecules only during prolonged inflammation *in vivo* ^32,33^. Further, recent findings indicate that astrocytes form immunological synapses with T cells, which are critical for the effectiveness of T cell response during infectious disease of the CNS^32^. Microglia also function as APCs that readily express MHC class II molecules upon activation^34^. However, no noticeable change was observed in IFN-γ expression upon microglia inactivation in the present study. These results imply that astrocytes function as APCs and contribute to Th1 cell activation. However, further studies need to be performed to determine the precise mechanisms underlying the effect of astrocytes on T cells.

At present, it is difficult to target T cells by using immunosuppressive and/or immunomodulatory drugs as a clinical treatment for patients with chronic pain. This is because most of these treatments have severe adverse effects such as hepatotoxicity and nephrotoxicity. Further, astrocytic inhibitors cannot be used in clinical practice because they are associated with severe neurotoxicity^35,36^. Therefore, an approach that targets the precise molecules involved in pain maintenance, such as IFN-γ, could be beneficial for treating central inflammation and might offer an absolute advantage of compromising immune function entirely.

In conclusion, our study suggests that inflammation triggers a positive feedback loop between astrocytes and Th1 cells through IFN-γ. Th1 cells and astrocytes act synergistically to promote the development of inflammatory pain.

## Methods

### Animals

Adult male Sprague–Dawley rats weighing 200–300 g (Experimental Animal Center, Fudan University School of Medicine, Shanghai, China) were housed in groups at a temperature- (22 °C ± 2 °C) and light-controlled (12-/12-h dark/light cycle) room with ad libitum access to food and water. Seven- to 10-week-old male wild-type mice and Rag1^−/−^ male mice on a C57BL/6j background were used for the experiments (The Jackson Laboratory). The experiments were performed in accordance with the guidelines of the Animal Care and Use approved by the Committee of Animal Experiments at Fudan University. All measures were taken to minimize the suffering of the animals and to reduce the number of animals used.

### Drug administration

LP injection was performed as described previously^11^. The rats were anesthetized using isoflurane and were placed on a plexiglass tube for widening the intervertebral spaces.

Drugs were delivered into the spinal space between the L5 and L6 vertebrae by using a 30-gauge needle. Instantaneous and rapid tail flick indicated a successful puncture. The following drugs (20 μl) were administered: minocycline hydrochloride (Sigma-Aldrich), fluorocitrate (Sigma-Aldrich), rat rIFN-γ (Peprotech), rat anti-IFN-γ monoclonal antibody (R&D systems), NS and 0.01 M PBS (pH 7.4).

### Induction of MA

CFA (50 μl and 20 μl; Sigma-Aldrich) was injected into the articular cavity of the unilateral ankle of animals (rats and mice, respectively) to induce MA. Surgeries were performed under isoflurane anaesthesia according to the procedures described previously^11^. In brief, the skin around the injection site was sterilized with iodine tincture and 75% alcohol. The left leg of the rat was held, and the fossa of the lateral malleolus of the fibula was located. A 28-gauge needle was inserted vertically to penetrate the skin and was turned distally to insert into the articular cavity from the gap between the tibiofibular and tarsus bone until a distinct loss of resistance was felt. Rats in the sham group were similarly injected with sterile NS.

### Primary astrocyte culture and drug treatments

Primary glial cultures were established from cells isolated from the cerebral hemispheres of 1- to 2-postnatal-day old Sprague–Dawley rats. After removing the meninges under a dissecting microscope, tissues were collected, centrifuged and treated with 0.25% trypsin–EDTA (Gibco) for 15 min at 37 °C. The reaction was terminated by adding DMEM/F12 (Gibco) supplemented with 10% foetal bovine serum (FBS; Gibco) and 1% penicillin/streptomycin. The cells were washed, resuspended in the culture media, plated in 75-cm^2^ flasks at a density of 1 × 10^5^ cells/ml and cultured at 37 °C in 5% CO_2_. After 2 weeks, astrocytes were obtained by shaking confluent primary mixed glial cell cultures. The cells were then plated in 6-well plates containing glass coverslips at a density of 1.5 × 10^5^ cells/ml. In subsequent experiments, the cells were incubated with the above culture media supplemented with 100 or 1000 U/ml rIFN-γ for 48 h.

### Von Frey test for mechanical pain hypersensitivity

Mechanical pain hypersensitivity was determined by measuring PWT in response to a calibrated series of Von Frey hairs (Stoelting) by using a protocol described previously^11^. In brief, rats were individually placed in a chamber (20 × 10 × 20 cm) containing a customized platform consisting of iron wires, which formed a 10-mm grid across the entire area. The animals were allowed to acclimate to the chamber for at least 30 min before performing the experiment. An ascending series of Von Frey hairs were applied to the mid-plantar surface of each hind paw. Each Von Frey hair was held for 2 s, with 15-s intervals between each application. A positive response was described as brisk withdrawal or paw flinching upon stimulation. When hind paw withdrawal was induced in at least 3 out of 5 consecutive applications of a particular filament, the value of that filament (in grams) was defined as PWT. The investigator was blinded to the group being tested. Mice were tested for mechanical allodynia using an electronic von Frey apparatus using filament #7 (IITC, Woodland Hills, CA). Three measurements were made for each time point and animal for mechanical hyperalgesia.

### Immunohistochemistry/Immunocytochemistry

Rats were deeply anaesthetized by injecting urethane (2 g/kg) intraperitoneally and were perfused intracardially with warm saline followed by 4% cold paraformaldehyde dissolved in 0.1 M phosphate buffer (pH 7.4). The L4–L5 segments of the spinal cord were removed, post-fixed in the same fixative for 4 h at 4 °C and passed through a 10–30% sucrose gradient. Transverse spinal sections (35 μm) were cut using a cryostat and were processed for immunofluorescence analysis. The sections were blocked with 10% donkey serum in 0.01 M PBS (pH 7.4) containing 0.3% Triton X-100 for 2 h at room temperature (RT). The sections were then incubated for 24–48 h at 4 °C with mouse anti-CD3 antibody (1:100 dilution; eBioscience), rabbit anti-IL-17 antibody (1:50 dilution; Santa Cruz Biotechnology), rabbit anti-IFN-γ antibody (1:100 dilution; Abcam), goat anti-Iba1 antibody (1:200 dilution; Abcam) or mouse anti-GFAP antibody (1:2000 dilution; Sigma) in 0.01 M PBS containing 1% normal donkey serum and 0.3% Triton X-100. After three 10-min washes, the sections were incubated with fluorescein isothiocyanate (FITC)-conjugated donkey anti-rabbit antibody (1:200 dilution; Jackson ImmunoResearch), FITC-conjugated donkey anti-goat antibody (1:200 dilution; Jackson ImmunoResearch), or rhodamine-conjugated donkey anti-mouse antibody (1:200 dilution; Jackson ImmunoResearch) for 120 min at 4 °C. The sections were examined under FV1000 confocal laser-scanning microscope (Olympus). Because activated glial cells were hypertrophic with processes, the area showing immunoreactive staining for Iba1 or GFAP in the ipsilateral superficial spinal dorsal horn (laminae I–III) was measured using Image-Pro Plus 6.0. The area of the ipsilateral superficial spinal dorsal horn was also calculated. The ratio of the above areas was used as the percentage area density of Iba1 or GFAP.

After 3 washes with 0.01 M PBS, cultured astrocytes grown on glass coverslips were blocked with 10% donkey serum in 0.01 M PBS containing 0.3% Triton X-100 for 30 min at RT. The cells were incubated overnight at 4 °C with rabbit anti-p-NF-κB p65 antibody specific to Ser-536 (1:500 dilution; Cell Signaling Technology) in 0.01 M PBS containing 1% normal donkey serum and 0.3% Triton X-100. The slides were washed with PBS and were incubated with rhodamine-conjugated donkey anti-rabbit secondary antibody (1:200 dilution; Jackson ImmunoResearch) before visualization under FV1000 confocal laser-scanning microscope. Optical density of immunoreactive staining for p-NF-κB p65 was measured using the Image-Pro Plus 6.0.

### Western blotting

After injecting an overdose of urethane, rat L4–L5 spinal segments were removed immediately, cut into ipsilateral and contralateral quadrants, frozen in liquid nitrogen and stored at −80 °C for further analysis. The samples were homogenized using an ultrasonic cell processor in SDS sample buffer containing proteinase inhibitors and PMSF. Equal amounts of the protein sample were separated by performing 10% tris–tricine SDS–PAGE and were transferred onto PVDF membranes (Millipore). The membranes were blocked with 5% nonfat milk for 2 h at RT. The membranes were incubated overnight at 4 °C with rabbit anti-IFN-γ antibody (1:500 dilution; Abcam), JNK (1:1000 dilution; Abcam), pJNK (1:1000 dilution; Abcam) or goat anti-GFAP antibody (1:10000 dilution; Sigma) and then with HRP-conjugated secondary antibodies (1:1000 dilution; Pierce) for 2 h at RT. Bands were developed using enhanced chemiluminescence (Pierce) and were visualized using ChemiDoc XRS system (Bio-Rad). All western blotting were performed at least 3 times, and similar results were obtained from all the analyses. The density of band area was quantified using Image Lab 3.0. A same-sized square was drawn around each band to measure its density, and the background of that band was subtracted. GAPDH expression was used as a loading control for protein expression.

### Flow cytometry

After injecting an overdose of urethane, the lumbar segments of rats were harvested, placed in 0.01 M PBS and homogenized into single-cell suspensions by using a cell strainer. Homogenates were washed with 0.01 M PBS, suspended in 30% and 70% discontinuous gradient Percoll (Sigma-Aldrich), and centrifuged at 390 × *g* for 30 min.

Mononuclear cells were collected, washed with 0.01 M PBS and resuspended in FACS buffer containing CD16/CD32 Fc-gamma RIII/RII-blocking antibody for 30 min at 4 °C. The cells were labelled with FITC-conjugated mouse anti-CD3 antibody (1:100 dilution; eBioscience) for 20 min at RT and were analysed using FACSCalibur with CellQuest software (Becton Dickinson). At least 10,000 cells were analysed per sample. Data are expressed as the percentage of CD3-positive cells among mononuclear cells.

### Statistical analysis

Data are expressed as mean ± standard error of the mean (SEM) and were obtained using Prism 5.0 (GraphPad). Behavioral data were analysed using two-way repeated measures (RM) analysis of variance (ANOVA), followed by Bonferroni post-tests. Data obtained from western blotting, immunofluorescence and flow cytometric analyses were analysed using Student’s *t*-test or one-way ANOVA with post-hoc Student–Newman–Keuls test. A p value of < 0.05 was considered statistically significant.

## Electronic supplementary material

Supplementary information
