# Supplementary material for: Bidirectional modulation between infiltrating CD3+ T-lymphocytes and astrocytes in the spinal cord drives the development of allodynia in monoarthritic rats
Source: Sci Rep. 2018 Jan 8;8:51. doi: 10.1038/s41598-017-18357-z (PMC5758647; doi:10.1038/s41598-017-18357-z)

**Bidirectional modulation between infiltrating CD3^+^ T-lymphocytes and astrocytes in the spinal cord drives the development of allodynia in monoarthritic rats**

Ya-Lan Zhou^1,+^, Shu-Zhuan Zhou^1,+^, Hao-Ling Li^1^, Man-Li Hu^2^, Hui Li^2^, Qing-Huan Guo^2^, Xiao-Ming Deng^1^, Yu-Qiu Zhang^2,*^, Hua Xu^1,*^

^1^Department of Anesthesiology, Changhai Hospital, The Second Military Medical University, Shanghai 200433, China

^2^Institutes of Brain Science and State Key Laboratory of Medical Neurobiology, Collaborative Innovation Center for Brain Science, Fudan University, Shanghai 200032, China

Ya-Lan Zhou: [yalanxzmc2007@126.com](mailto:yalanxzmc2007@126.com)

Shu-Zhuan Zhou: [zszmenji188@qq.com](mailto:zszmenji188@qq.com)

Hao-Ling Li：[539924641@qq.com](mailto:539924641@qq.com)

Man-Li Hu: [15211520008@fudan.edu.cn](mailto:15211520008@fudan.edu.cn)

Hui Li: [10210700027@fudan.edu.cn](mailto:10210700027@fudan.edu.cn)

Qing-Huan Guo: [11210700019@fudan.edu.cn](mailto:11210700019@fudan.edu.cn)

Xiao-Ming Deng: [deng_x@yahoo.com](mailto:deng_x@yahoo.com)

^+^These authors contributed equally to this work.

^*^Correspondence and requests for materials should be addressed to Yu-Qiu Zhang (email: [yuqiuzhang@fudan.edu.cn](mailto:yuqiuzhang@fudan.edu.cn)) or Hua Xu (email: [pshhuaxu@163.com](mailto:pshhuaxu@163.com))

Supplementary Figure legends

Figure 1. joint-swelling on day 10 post-MA.

Figure 2. Full length blots of those cropped and presented in the main figures.


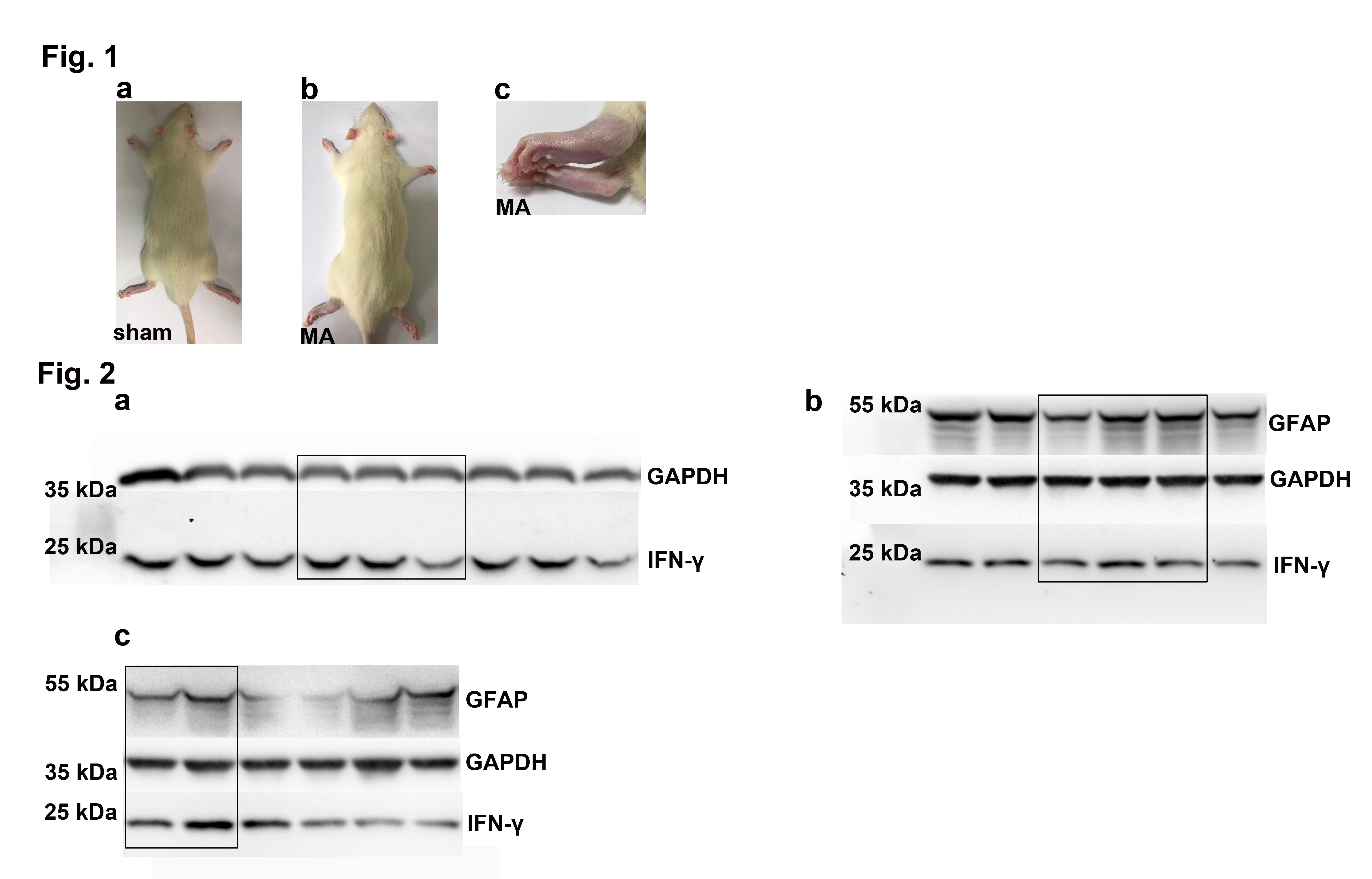

Supplement: Supplementary file 1 — Supplementary information [file 41598_2017_18357_MOESM1_ESM.doc]
